# Supplementary material for: Should my automated car drive as I do? Investigating speed preferences of drivengers in various driving conditions
Source: PLoS One. 2023 Feb 9;18(2):e0281702. doi: 10.1371/journal.pone.0281702 (PMC9910714; doi:10.1371/journal.pone.0281702)
Supplement: Attachment — Submitted filename: Response to Reviewers.docx [file pone.0281702.s002.docx]

| **REVIEWER #1** | | |
| --- | --- | --- |
| **#** | **Question/comment** | **Authors’ answer** |
| 1 | *In the present study, the authors investigated the drivers' preferences for an SAE 3rd-level AV over different traffic conditions. After reviewing the manuscript, the following are my comments.* | The authors thank you very much for the time spent reviewing our manuscript and for your precious feedback.  All changes made in response to your suggestions are highlighted in yellow in the manuscript. |
| 2 | *In the introduction section, the authors listed the outcomes from the previous studies. But as a reader, it is essential to understand the research needs for carrying out the present work. I recommend that the authors dig into the research gaps and highlight the need for carrying out this work.* | As suggested, additional background information has been added to make the research needs for this study clearer to the reader (lines 55-56, 58-59 and 107-108). |
| 3 | *In the present case, authors used 24 scenarios for testing for an SAE 3rd-level AV. But in the current work, it needs to be clarified how those scenarios are selected and how they are adapted and validated is not clear. Further, how they imitate the SAE 3rd Level compared to other SAE level vehicles.* | The authors apologize if these points were not clear during your review.  The scenarios were not selected, but constructed for the study (this is specified on line 203). The variables used in the scenarios were selected because they are characteristic of the driving activity, and determine its complexity (this is specified on lines 121-122). The validity of the use of this type of scenarios comes from previous studies conducted using the same method (this is specified in lines 199-200).  Finally, what allowed participants to imagine being in an SAE Level 3 automated vehicle (and not in another level) was the description given during the instruction phase. Indeed, the characteristics of this level of automation were described at that time and then recalled at the end of the familiarization phase (this is specified in the Scenario instructions section). |
| 4 | *Further, I recommend that the authors present some pictorial representation of the scenarios. In the present form, the description is very brief, with limited details.* | The authors apologize if this was confusing, but the scenarios used were only written. A complete example of a scenario is shown on lines 206-207. The situations described are deliberately not very detailed, as it allows for a specific focus on the effect of certain variables of interest without adding too much noise. This also comes with limitations, which is discussed in the limitations of the study on lines 499-506. |
| 5 | *The title says, "Should my automated car drive as I do" but I didn't detail how the authors ascertained the human driver behaviour for comparing with automated driving.* | In the context of this study, the anticipatory behavior of human drivers was assessed by the speed reported by the participants for each condition. This speed was then compared to their reported preferred speed for automated driving in the respective conditions. This is specified in lines 212-217. |
| 6 | *I recommend that the authors present a research methodology section to explain the flow of the work. In the present form, it is tough to understand the flow of the work.* | The authors apologize if it was difficult to understand the flow of the work. We have made a minor change to the Procedure section (line 242), which from our perspective already has the function of the section you suggest be added to the manuscript. This section outlines the various stages of the study, from the first click on the study link to the last question in the study. |

| **REVIEWER #2** | | |
| --- | --- | --- |
| **#** | **Question/comment** | **Authors’ answer** |
| 1 | *The current study aimed to investigate the benefits of automated driving styles personalization hold in various driving conditions.* | The authors thank you very much for the time spent reviewing our manuscript. |
| 2 | *The paper is not well written. As an example, the expectation is that the authors state the literature review and then state the objective and contribution of their work based on that. However, when they reach the objective expression, they have to write another part of literature review (i.e., lines 120-160). This was just an example; but here are more in the manuscript.* | The authors are sorry that you do not find the manuscript well written. We have put a lot of effort into making it as readable as possible. The introduction, as well as the rest of the manuscript, follows the submission guidelines proposed by the journal. |
| 3 | *Sampling has a direct impact on the accuracy of interpretation of the results and its application. How did the authors of the article ensure that the number of 103 drivers with the stated characteristics can be representative of the community? Given how they were selected, how was it ensured that the selection was not biased?* | The authors agree that sampling is an important part of a study, and has a direct impact on the accuracy and results and its application.  In the context of this study, we made sure to recruit participants with the most diverse profiles possible. This was not done by using a standardized method, but by distributing the call for participants to various audiences (e.g., retired persons, students, etc.).  The effectiveness of this method is reflected in the characteristics observed in our sample: there is a very good distribution of the participants' biological sex, age, car use and experience with automated driving systems. We therefore believe that the sample selected does not bias the results highlighted in the study.  Beyond that, we made a point of ensuring that our results have high internal validity, by controlling for the statistical power of each hypothesis test. |
| 4 | *As the authors of the article themselves have acknowledged, such studies have been based on either real road experiments or in a driving simulator. Using the scenario and participants' answers, although it may provide the possibility of asking various questions, but its validation is very crucial; especially when people's behavior is examined. How can the authors of this research ensure that the participants in reality show the same behavior as they said in response to the scenarios far from the conditions? I read the Procedure section but still doubt.* | The authors agree that the question of external validity is a central issue for this type of research. It is important to note that this limitation is discussed on lines 499-506.  Furthermore, the issue of the relationship between self-reported behavior and driving simulator or real road behavior has been studied before, and one of the main references is cited in lines 169-171. This study shows that self-reported speed can be used to replace direct observation (i.e., on real road).  However, the authors agree that validation of the results shown in this study on a driving simulator or on a real road would be a relevant next step (as discussed on lines 505-506). We believe that science is a cumulative process, and that this study paves the way for many other experiments. |
| 5 | *The article has several hypotheses and has been concluded with a very simple statistical analysis. I don't see a strong methodology or proper modeling in this paper. This is a major weakness for this research and publication in such a quality journal.* | With regard to the theoretical models considered in the manuscript and past studies on the subject, we formulated several hypotheses. We believe that these have all been tested with appropriate statistical tests.  Regarding the methodology used, as discussed in the previous answers, it is one of the validated alternatives to driving simulator and real road experiments. |

| **REVIEWER #3** | | |
| --- | --- | --- |
| **#** | **Question/comment** | **Authors’ answer** |
| 1 | *This study investigates the extent to which speed personalization of automated driving styles is desirable with respect to various driving conditions and trust in an SAE level 3 automated car. The research gap has been clearly defined. Further, authors have presented and discussed the experiment design and study outcomes thoroughly.* | The authors thank you very much for the time spent reviewing our manuscript and for your positive feedback. |

| **REVIEWER #4** | | |
| --- | --- | --- |
| **#** | **Question/comment** | **Authors’ answer** |
| 1 | The authors present a clear and focused study that is well placed among the literature. To my knowledge (which I consider to be intermediate), the methods seem appropriate. The story builds logically to the conclusions. The authors state the limitations and suggest relevant future work. | The authors thank you for the very high quality of your review, and the interest shown in our manuscript. |
| 2 | The underlying data is probably available on the OSF website, but I don’t have access to confirm. | As suggested, the problem has been corrected. Access to the data is now public. |
| 3 | I suggest some minor edits in the attached file. A few comments ask for further explanation. Most comments are to help the authors to increase the readability. Note that some comments start with ‘Consider’. Their suggestions are optional, but I suggest them to improve readability according to my preferences. Authors, please use your judgement about the audience’s preferences. | Thank you very much for your valuable and detailed feedback on the manuscript.  Below, we answer the main questions/comments you left on the document.  All changes made in response to your suggestions are highlighted in green in the manuscript. This covers the main questions/comments you left on the document as well as other points raised (e.g., editorial elements). |
| 3.1 | [About the abstract]  *If you have room, please state the methods and statistical tests that you applied. A list is sufficient if you lack space.* | As suggested, the methods and statistical tests applied are now stated in the abstract at lines 36 and 40-43. |
| 3.2 | [About the bold part in this sentence: Keeping in the comfort zone regarding progress implies maintaining speed and fluent progress, **such that deceleration can be felt punishing and keeping the speed and pace represent the comfort zone.**]  *Please revise. I'm unclear on the meaning of this part of the sentence.* | As suggested, the sentence has been reworded to be as clear as possible (lines 75-78). |
| 3.3 | [About the use of “driving styles”]  *Consider:*  *- whether you need the s, and*  *- hyphenating driving style to avoid confusion,*  *giving driving-style.* | We have decided to follow your recommendation of hyphenating driving style (giving *driving-style*) in order to avoid confusion. This change has been applied to the whole document. |
| 3.4 | [About the use of single-sentence paragraphs]  *I believe that a paragraph should contain at least two sentences. Consider merging these last two paragraphs.* | As suggested, some paragraphs have been merged (e.g., the paragraph starting at line 166). |
| 3.5 | [About the interpretation of statistical power analyses]  *Please add 1-2 sentences to interpret the results (perhaps using an example) for the reader. What do the results mean for practice or for your conclusions? What criteria do the results satisfy?* | As suggested, some elements of interpretation of the results have been added. However, the results of the sensitivity analyses should be interpreted in relation to the effect sizes observed through analysis of the data, in order to identify potential sources of bias or uncertainty in the results. That is why this point is being raised in the Discussion section. |
| 3.6 | [About the bold part in this sentence: Taken together, results for **hypotheses 3** and H4 suggest that trust in ACs could play an important role in the desirability of an automated driving system with personalized speed.]  *Should this term be H3?* | Thank you for your suggestion. We finally decided to keep the term like this, since there are several sub-hypotheses 3, unlike H4 which is a single hypothesis. |
| 3.7 | *Overall, the paper is good. I enjoyed reading and reviewing it. Well done!* | Again, thank you very much for the time spent reviewing our manuscript. |
